# Research on Motor Neuron Diseases Konzo and Neurolathyrism: Trends from 1990 to 2010

**DOI:** 10.1371/journal.pntd.0001759

**Published:** 2012-07-31

**Authors:** Delphin Diasolua Ngudi, Yu-Haey Kuo, Marc Van Montagu, Fernand Lambein

**Affiliations:** 1 Programme National de Nutrition (PRONANUT), Kinshasa, Democratic Republic of the Congo; 2 Institute of Plant Biotechnology Outreach (IPBO), Ghent University, Ghent, Belgium; Institute of Tropical Medicine, Belgium

## Abstract

Konzo (caused by consumption of improperly processed cassava, *Manihot esculenta*) and neurolathyrism (caused by prolonged overconsumption of grass pea, *Lathyrus sativus*) are two distinct non-infectious upper motor neurone diseases with identical clinical symptoms of spastic paraparesis of the legs. They affect many thousands of people among the poor in the remote rural areas in the central and southern parts of Africa afflicting them with konzo in Ethiopia and in the Indian sub-continent with neurolathyrism. Both diseases are toxico-nutritional problems due to monotonous consumption of starchy cassava roots or protein-rich grass pea seeds as a staple, especially during drought and famine periods. Both foods contain toxic metabolites (cyanogenic glycosides in cassava and the neuro-excitatory amino acid β-ODAP in grass pea) that are blamed for theses diseases. The etiology is also linked to the deficiency in the essential sulfur amino acids that protect against oxidative stress. The two diseases are not considered reportable by the World Health Organization (WHO) and only estimated numbers can be found. This paper analyzes research performance and determines scientific interest in konzo and neurolathyrism. A literature search of over 21 years (from 1990 to 2010) shows that in terms of scientific publications there is little interest in these neglected motorneurone diseases konzo and neurolathyrism that paralyze the legs. Comparison is made with HTLV-1/TSP, an infectious disease occurring mainly in Latin America of which the clinical manifestation is similar to konzo and neurolathyrism and requires a differential diagnosis. Our findings emphasize the multidisciplinary nature of studies on these neglected diseases, which however have not really captured the attention of decision makers and project planners, especially when compared with the infectious HTLV-1/TSP. Konzo and neurolathyrism can be prevented by a balanced diet.

## Author Summary

The irreversible crippling diseases konzo and neurolathyrism with identical clinical symptoms occur among poor subsistence farmers in Africa and Asia. The victims are mostly illiterate and among the poorest section of the population who can only afford the cheapest food in a monotonous diet: bitter cassava roots (*Manihot esculenta*) in the case of konzo, or the seeds of grass pea (*Lathyrus sativus*) in the case of neurolathyrism. These neurodegenerations are blamed on the presence of cyanogenic glycosides in cassava and a neuro-excitatory amino acid in grass pea. Both are linked to the deficiency of methionine and cysteine in the diet. These amino acids are needed for the protection of motor neurons against oxidative stress. Both cassava and grass pea are tolerant to adverse environments and become survival foods for the poor during drought-triggered famines and during wartime. The dependency on these crops may increase with global warming. The scientific interest in these neglected diseases is low and little change has been noted over the last two decades. This is especially obvious when compared with the scientific interest in HTLV-1/TSP, a viral disease with similar clinical symptoms which occurs mainly among poor people in Latin America in similar socio-economic settings.

## Introduction

Konzo and neurolathyrism are toxico-nutritional neurodegenerations that afflict many thousands of people among poor populations in low income countries, especially in Sub Saharan Africa and in the Indian Sub-continent, respectively. In both cases, the victims are mainly illiterate subsistence farmers living in remote rural areas. Those diseases are associated with high dietary exposure from improperly processed cassava roots in the case of konzo or grass pea seeds in the case of neurolathyrism. Both cassava (*Manihot esculenta*) and grass pea (*Lathyrus sativus*) are drought tolerant crops that can be survival food after a drought (the underground cassava-roots can even survive fire) [1]. Cyanogenic glycosides linamarin and lotaustralin in cassava and the neuro-excitatory amino acid β-ODAP (β-N-oxalyl-α,β-diaminopropionic acid) in grass pea are substances incriminated in the causation of konzo and neurolathyrism respectively, and to be involved in the depletion of the essential sulfur amino acids provided by the diet. Both foods are deficient in the sulfur containing amino acids methionine and cysteine. These amino acids are required for the detoxification of the cassava cyanogens by the liver enzyme rhodanese [2]. Besides ODAP, a number of anti-nutritional factors present in grass pea as in all legume seeds can also contribute to depleting the sulfur containing amino acids that are essential for the maintenance of the redox homeostasis in the body [3]. The depletion of these amino acids in the body by prolonged consumption of cassava roots or grass pea seeds as staple contributes to oxidative stress.

Clinically, both diseases cannot be differentiated as both are motor neurone diseases with symmetric spastic paraparesis of the legs, an irreversible crippling disability presenting in different stages of severity [4]. Both diseases can be prevented by means of good household food security, availability of safe water, good food practices and handling, balanced and varied diets, and good health status, factors that are in jeopardy during droughts. Fortunately, these two diseases have not yet been reported to occur in the same geographical area. If so, it would be difficult to distinguish one from the other although there are some epidemiological differences. The patient's history with the consumption of either cassava or grass pea is needed for the diagnosis. Both diseases are characterized by sudden onset, with higher incidence during the dry season for konzo or after a drought when other crops except grass pea fail for neurolathyrism. However, the most affected group is different (mainly young children and women at child-bearing age for konzo, mainly young men for neurolathyrism). From the available literature, we can hypothesize that both diseases can occur in epidemics, when three main risk factors occur simultaneously: i) extreme poverty, ii) a high degree of illiteracy and iii) the availability of bitter cassava roots in the case of konzo, or grass pea seed in the case of neurolathyrism, as the cheapest food available. In addition, heavy physical labor was also identified as an epidemiological risk factor in both diseases. The sudden occurrence of konzo in young mothers after childbirth is particularly tragic.

The poorest sections of the population are often illiterate, have little or no political voice and are the most vulnerable for konzo and neurolathyrism. As an apparent consequence, fighting these diseases is of low priority for decision makers or governments. Both diseases involve the irreversible loss of motor neurons for which there is no cure. Some researchers have tried symptomatic treatment with a muscle relaxant ‘Tolperisone’ for neurolathyrism patients. Although the mobility of the less severely affected patients improved, this treatment was not put into practice because of the high cost [5], [6]. Small scale efforts have been made on improving the methods for reducing cyanogens during post-harvest processing of cassava [7] or for reducing β-ODAP during culinary preparation of grass pea [8] but until now these methods could not prevent the occurrence of these diseases. To our knowledge no comprehensive epidemiological studies have yet been conducted or planned. There are no accurate numbers of cases since both diseases are not considered reportable by the World Health Organization (WHO). Poverty and illiteracy of the victims, the knowledge that there is no cure and remoteness of the areas of incidence make underreporting common. There are few reliable statistics on the prevalence of both diseases and at best only estimations have been made. Little attention has been given by governments in afflicted countries [2]. As a consequence, financial support for research on the etiology of these diseases and their prevention has been insufficient and erratic.

The aim of this paper is to analyze research performance and to determine scientific interest in konzo and neurolathyrism in terms of the number of quality articles that have been published over 21 years since the first scientific article [9] that recognized konzo as a distinct disease entity was published in the British journal “Brain”. We also studied the scientific interest in these diseases by analyzing the trend in citation frequency of the scientific articles by other researchers. The publication and citation performance of these toxico-nutritional diseases was compared with the ones of the infectious disease HTLV-1 associated myelopathy/Tropical spastic paraparesis (HAM/TSP). The clinical manifestation of HAM/TSP is similar to konzo and neurolathyrism and this neurological disease has been considered for differential diagnosis with konzo and neurolathyrism. The three diseases occur in similar socio-economic settings. As in konzo and neurolathyrism, environmental factors and geographic locations seem to be important in the etiology of Tropical Spastic Paraparesis [10]. Many cases of TSP are not linked to HTLV-1 infection and some authors consider konzo and neurolathyrism as variants of Tropical Spastic Paraparesis [11].

## Methods

We searched the Web of Science database (http://apps.isiknowledge.com/) for all articles with the following key words: *konzo*, *neurolathyrism*, *HTLV-1 associated myelopathy/Tropical spastic paraparesis* in topic and in title with the current limits on timespan (from 1990 to 2010). The term ‘lathyrism’ covers both neurolathyrism and osteolathyrism. Osteolathyrism or experimental lathyrism is caused by the bone-deforming β-aminopropionitrile (BAPN) present in *Lathyrus odoratus* [12]. Because of this ambiguity we have screened those papers on ‘lathyrism’ and the papers dealing with the neurological affection were classified under ‘neurolathyrism’. The results were refined by subject areas, document types, publication years and times cited. No restriction was applied for language and subject areas while correction material was excluded from the document types and the year 2011 was not considered for the citation report. We accessed also The PubMed database (http://www.ncbi.nlm.nih.gov/sites/entrez?otool=ibeuglib) but some overlap was noted on the categorization of the document types and the database has a medical orientation rather than multidisciplinary as in the Web of Science. The website http://Scholar.Google.com was not included because of a great number of subjects which comprise even unpublished or non-quoted publications. Articles listed in Web of Science are classified as A1 papers and used for evaluation purposes in many scientific institutes and universities.

Data were introduced in software package Microsoft Excel 2010 and SPSS 17 to calculate average and proportions, to draw tables and graphics, to analyze regression by means of curve estimation, scatter plots with simple linear regression were displayed for time series and R-squared were calculated to measure how well the regression line approximates real data points. The independent variable was year of publication, ANOVA table was displayed and a P-value of <0.05 was considered statistically significant.

## Results

**Figure 1:**
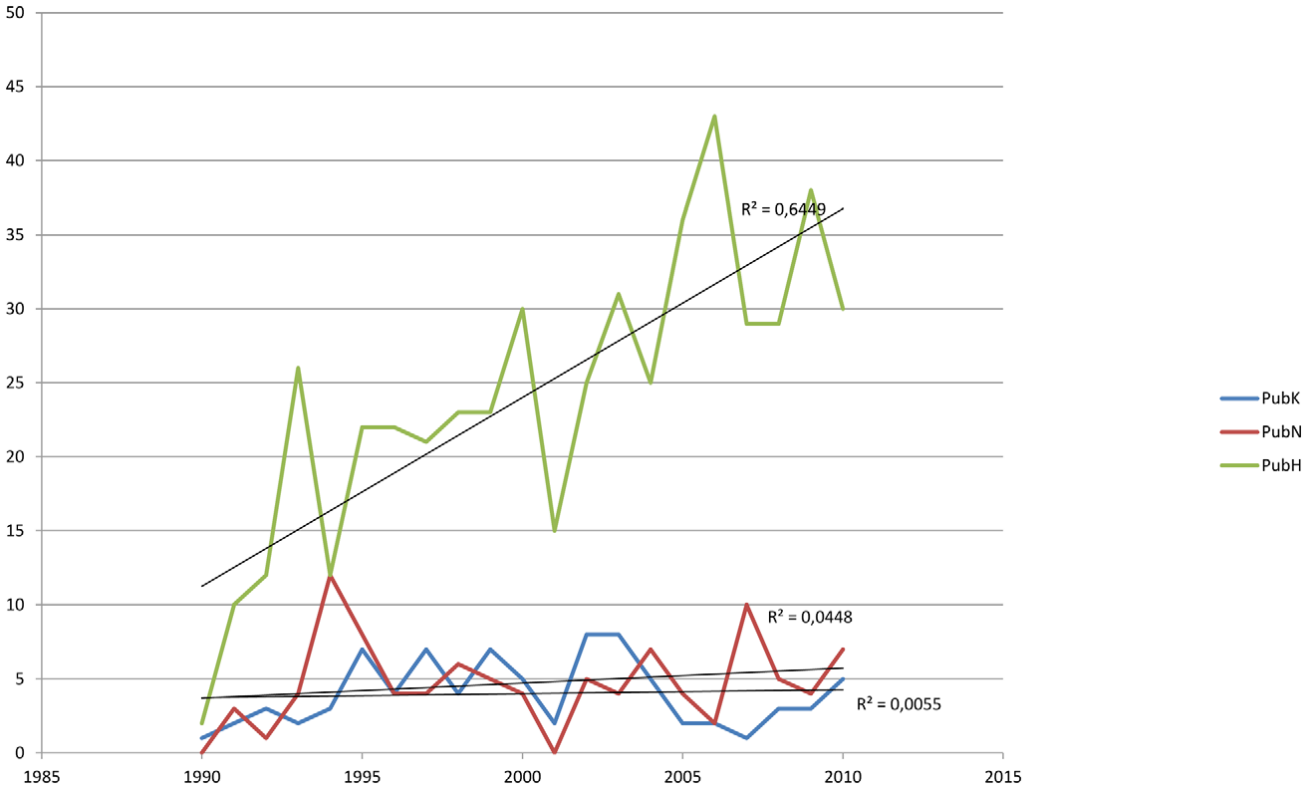
Trend in the number of publications on Konzo (R^2^ = 0.006; P = 0.749), Neurolathyrism (R^2^ = 0.045; P = 0.357) and HAM/TSP (R^2^ = 0.645; P = 0.001) during 21 years.

**Figure 2:**
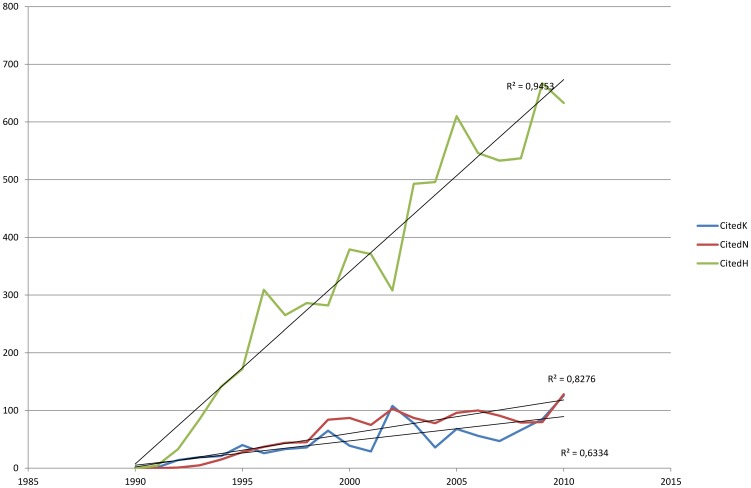
Trend in number of citations per year to original papers on Konzo (K), (R^2^ = 0.633; P = 0.001), Neurolathyrism (N) (R^2^ = 0.828; P = 0.001) and HTLV-1 associated myelopathy/Tropical Spastic Paraparesis (H) (R^2^ = 0.945; P = 0.001).

Since the first publication which recognized konzo as a distinct disease entity in 1990 till the end of December 2010, our search in the Web of Science yielded 84, 99 and 504 items published on konzo, neurolathyrism and on HAM/TSP respectively. This represents an average of 4 publications per year for konzo topics, of 5 publications for neurolathyrism and of 25 publications for HTLV-1/TSP topics. The terms konzo, neurolathyrism and HTLV-1/TSP were found in the title of 39.3%, 34.3% and 14.9% respectively of these published topics. Figure 1 shows that there was no significant linear increase over time in the total number of publications on konzo (R^2^ = 0.006; P = 0.749) and neurolathyrism (R^2^ = 0.045; P = 0.357) while publications on HTLV-1/TSP increased significantly (R^2^ = 0.645; P<0.05). A significant increase (P<0.05) was observed in the citations of publications on the three diseases (Figure 2).

**Figure 3:**
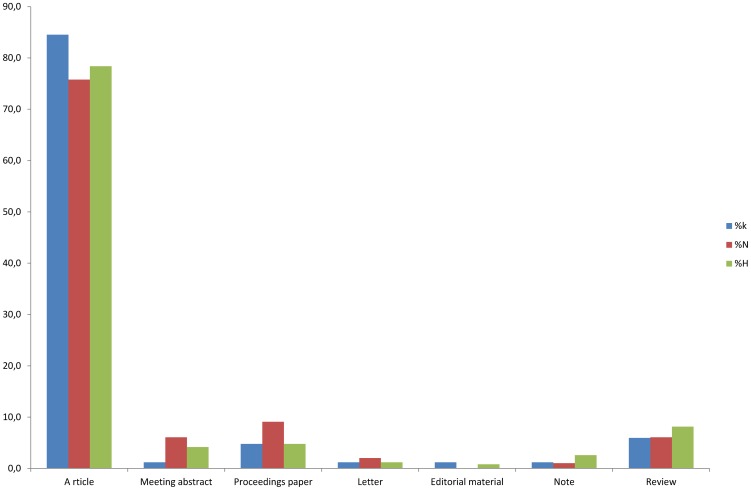
Percentage of document types published in the area of Konzo (k), Neurolathyrism (N) and HAM/TSP (H).

When published items were analyzed per document type, more than 75% of these publications (84.5% for konzo; 75.8% for neurolathyrism and 78.4% for HTLV-1/TSP) were categorized as articles, about 6% for konzo and neurolathyrism and 8% for HTLV-1/TSP as reviews, 4.8% for konzo and HTLV-1/TSP and 9.1% for neurolathyrism as proceedings paper, 1.2% for konzo, 6.1% for neurolathyrism and 4.2% for HTLV-1/TSP as meeting abstract and 2% or less as letter or as note or as editorial material (Figure 3).

We compared the keywords used for konzo and neurolathyrism items. The top three of the most frequently used terms are konzo, cassava and cyanide for konzo publications and neurolathyrism, *Lathyrus sativus* and β-ODAP for neurolathyrism publications. Motor neurone disease is the only term used for these two clinically similar diseases among the top ten keywords (Table 1).

**Table 1:** Top 10 of the most frequently used author keywords in papers on konzo and neurolathyrism.

Konzo (#)	Neurolathyrism (#)
*1. Konzo (32)*	*1. Neurolathyrism (42)*
*2. Cassava (25)*	*2. Lathyrus sativus (24)*
*3. Cyanide (19)*	*3. β- ODAP (22)*
*4. Linamarin (8)*	*4. Neurotoxin (10)*
*5. Spastic paraparesis (8)*	*5. Motor neuron disease (10)*
*6. Manihot esculenta (6)*	*6. Grass pea (7)*
*7. Cyanogens (6)*	*7. Excitotoxicity (7)*
*8. Thiocyanate (6)*	*8. Oxidative stress (7)*
*9. Motor neuron disease (5)*	*9. Excitatory amino acids (6)*
*10. Cyanogenesis (4)*	*10. BOAA (5)*

**Figure 4:**
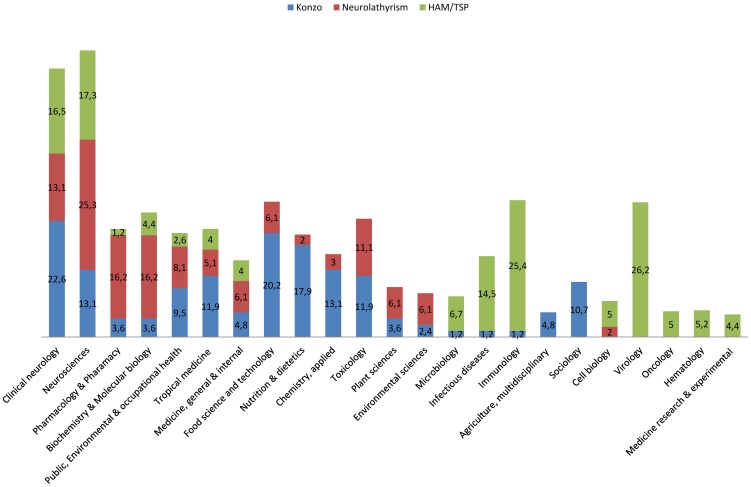
Distribution of papers on Konzo, Neurolathyrism and HAM/TSP in journals in various subject areas.

Concerning the subject areas of these publications, we identified 23 areas (see Figure 4) as the most frequently cited, of which 7 are related to publications on the three diseases (Clinical Neurology, Neurosciences, Pharmacology & Pharmacy, Biochemistry & Molecular Biology, Public Environmental & Occupational Health, Tropical Medicine, Medicine General & Internal), 6 to konzo and neurolathyrism (Food Science & Technology, Nutrition & Dietetics, Applied Chemistry, Toxicology, Plant Sciences, and Environmental Sciences), 3 to konzo and HTLV-1/TSP (Microbiology, Infectious Diseases and Immunology), 1 to neurolathyrism and HTLV-1/TSP (Cell Biology), 2 to konzo (Agriculture, Multidisciplinary and Sociology) and 4 to HTLV-1/TSP (Virology, Oncology, Hematology and Medicine Research & Experimental). Clinical Neurology (22.6%), Food Science & Technology (20.2%), Nutrition & Dietetics (17.9%), Neurosciences (13.1%) and Applied Chemistry (13.1%) are the main areas in konzo publications while Neurosciences (25.3%), Biochemistry & Molecular Biology (16.2%), Pharmacology & Pharmacy (16.2%), Clinical Neurology (13.1%) and Toxicology (11.1%) are the main areas in neurolathyrism publications, whereas Virology (26.2%), Immunology (25.4%), Neurosciences (17.3%), Clinical Neurology (16.5%) and Infectious Diseases (14.5%) are the main areas in the HTLV-1/TSP publications.

## Discussion

Our search in the Web of Science shows that over 21 years the number of items published on konzo and neurolathyrism has increased slowly but not significantly when compared to the significant increase in studies on HTLV-1/TSP. HTLV-1/TSP is less hidden from world view because it mainly occurs in Latin America, a higher profile area, whereas the others occur largely in remote parts of sub-Saharan Africa and dry, remote parts of India amongst very poor people. Even in the African countries where konzo and neurolathyrism occur, they are largely hidden diseases, which are (in the case of konzo) often considered to be due to witchcraft. They are neglected and tend even to be ignored by the authorities in these countries, perhaps because they are somehow considered as damaging the reputation of the country in which they occur. HTLV-1/TSP is intrinsically more interesting to medical researchers because it is an infectious disease that has the risk of spreading, whereas neurolathyrism and konzo are non-infectious diseases that are confined to poor and mostly illiterate subsistence farmers and hence not appealing to work on. HTLV-1 TSP has a higher percentage of reviews and notes than konzo and neurolmathyrism as shown in Figure 3 which indicates more interest and funds for research in this area.

This also reflects the low level of interest by political authorities as well as by institutes sponsoring research in these neglected diseases konzo and neurolathyrism which are known to be non-infectious diseases affecting only poor populations in remote rural areas. These diseases do not really capture the attention of decision makers and project planners to make efforts to control these diseases while there is a growing interest and concern in the infectious HTLV-1/TSP.

During the same period there is an increase of the number of citations to published items on the three diseases, potentially indicating that those diseases are becoming important in terms of comparison with neurodegenerative diseases affecting more affluent populations. It may be interesting to note that, not unlike the popular media, the dramatic reports get more attention: the report on a new epidemic in Ethiopia, affecting 2000 patients [13], received 3.92 citations per year; while elaboration of the risk and protective factors that may offer a key to prevention, published in the same journal [14], received far less attention (2.11 citations per year).

There are fewer meetings at international level on those three diseases, which may explain why there is less than 10% of meeting abstracts or proceedings papers. Sponsors may not be well informed or do not see the opportunities to finance projects on konzo and neurolathyrism although our findings emphasize the multidisciplinary nature of studies on these diseases.

**Figure 5:**
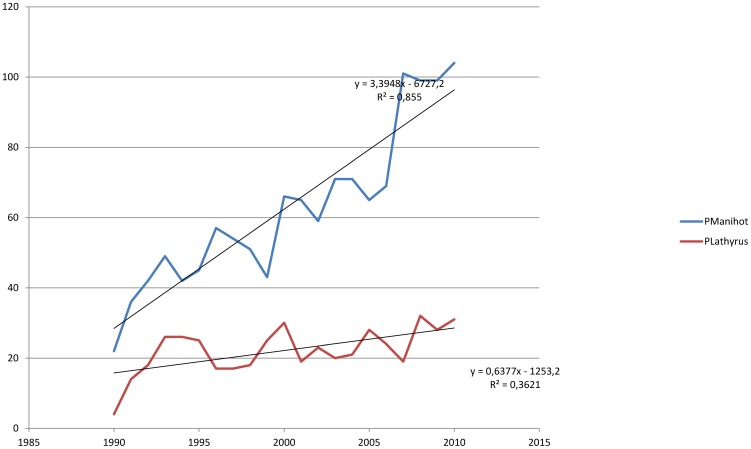
Evolution of the number of papers per year on the plants incriminated as cause of Konzo (*Manihot esculenta*) and Neurolathyrism (*Lathyrus sativus*).

While the two neglected diseases konzo and neurolathyrism receive a comparable low level of attention in scientific publications, there is a great difference regarding to the plants incriminated as causes of these diseases. The number of papers dealing with cassava or *Manihot esculenta* during the same period (5035) is almost 6 times greater than those dealing with grass pea or *Lathyrus sativus* (859) (Figure 5). This rapid growth in papers on cassava is undoubtedly due to its great importance as a world food source particularly in tropical Africa where it is the staple food. Also the industrial use of cassava roots for the production of chicken pellets, starch and biofuel attracts much more studies when compared with grass pea, although grass pea produces the cheapest dietary protein in arid areas of East Africa and the Indian sub-continent.

The root of bitter cassava is the staple food in konzo-affected areas and is one of the highest yielding starch crops in tropical regions and increasingly becoming an industrial product. For human consumption, a lengthy post harvest processing is necessary to remove the toxic cyanogenic metabolites. For this a stable peaceful environment is required. This processing however does not change the deficiency of the sulfur amino acids methionine and cysteine in the cassava roots. While one meal of unprocessed bitter cassava roots can be lethal and indeed sometimes suicidal, prolonged consumption of insufficiently processed roots as a staple food without variation can give rise to konzo. Konzo can be prevented by better processing of bitter cassava roots and by balancing the diet with cereals and other foodstuffs rich in sulfur amino acids such as fish that is out of reach of the very poor and during social instability. For poor subsistence farmers, cassava can be a survival food during drought and also during military conflicts. Banea et al [15] showed recently that konzo can be prevented in village people by using the wetting method on cassava flour, resulting in an extra reduction of cyanogen intake.

Grass pea is the most drought tolerant legume producing the cheapest protein, but containing a neuro-excitatory amino acid β-ODAP and can give rise to excito-toxicity under certain conditions of prolonged overconsumption, malnutrition and oxidative stress. This same neuro-excitatory amino acid β-ODAP is also identified in seeds and roots of Ginseng (*Panax ginseng*) [16]. In Chinese traditional medicine Ginseng root is considered a longevity promoting substance. The haemostatic compound “dencichine” extracted from Ginseng is identical to β-ODAP and has been patented as a herbal medicine [17]. Moderate daily consumption of grass pea like other legumes has no deleterious effects, and some authors even mention beneficial effects for human health [18]. However, neurolathyrism often occurs as a consequence of drought-triggered famine. It does not affect longevity or cognitive functions, but the patients become dependent on already deprived and often neglected remote rural communities. The research focus on toxic aspects such as appeared in clinical neurology, neurosciences, pharmacology and toxicology may have contributed to the toxic reputation of *Lathyrus sativus*, that during the era of the Egyptian pharaoh's was a royal funeral offering. Konzo and neurolathyrism are neglected diseases that occur among poor, often illiterate subsistence farmers. Such socio-economic groups are often neglected by the authorities and these medical problems ignored. Konzo and neurolathyrism can be considered the emanation of an unjust and egoistic world.

### Future directions

One of the risk factors in the epidemiology of konzo and neurolathyrism is the availability of cassava roots or grass pea as the cheapest food and its use as staple in the diet with little or no additional nutrients. Both crops are tolerant to adverse environment and serve as survival foods during droughts and famine and this dependency may increase with further global warming [19]. Both konzo and neurolathyrism can be linked to low concentrations of plasma methionine as a result of a dietary deficiency of this amino acid [20]. As a result, the reduced level of glutathione jeopardises the defense of motor neurons against oxidative stress. Creating market conditions for making alternative food available that is cheaper than cassava roots or grass pea with a better balance of essential amino acids can be a key strategy in the prevention of konzo and neurolathyrism. Genetic improvement of both crops should be aimed at increasing the level of the essential sulfur amino acids methionine and cysteine as long term prevention. This view is recently supported by Ethiopian researchers for the nutritional improvement of grass pea [19], while other authors also document the need for renewed impetus in grass pea research [21]. The present regulation of genetically modified food crops makes the cost for developing more healthy cassava or grass pea varieties exorbitant and contributes to the neglect of these preventable diseases.
